# Eucalypt leaf litter impairs growth and development of amphibian larvae, inhibits their antipredator responses and alters their physiology

**DOI:** 10.1093/conphys/coy066

**Published:** 2018-12-10

**Authors:** Pablo Burraco, Maider Iglesias-Carrasco, Carlos Cabido, Ivan Gomez-Mestre

**Affiliations:** 1 Ecology, Evolution and Development Group, Doñana Biological Station (CSIC), C/ Americo Vespucio 26, Sevilla, Spain; 2 Evolutionary Biology Centre, Uppsala University Norbyvägen 18 D, Uppsala, Sweden; 3 Department of Evolutionary Ecology, National Museum of Natural History (CSIC), Calle de José Gutiérrez Abascal, 2, Madrid, Spain; 4 Department of Herpetology, Aranzadi Society of Sciences, Zorroagagaina, 11, San Sebastian, Spain; 5 Research School of Biology, Australian National University, 134, Linnaeus Way, Acton ACT Canberra, ACT, Australia

**Keywords:** Amphibians, exotic plants, global warming, immune response, metabolic rate, oxidative stress, predators

## Abstract

Consequences of human actions like global warming, spread of exotic species or resource consumption are pushing species to extinction. Even species considered to be at low extinction risk often show signs of local declines. Here, we evaluate the impact of eucalypt plantations, the best-known exotic tree species worldwide and its interaction with temperature and predators on amphibian development, growth, antipredator responses and physiology. For this purpose, we applied a fully factorial experiment crossing two types of leaf litter (native oak or eucalypt), two temperatures (15 and 20°C) and presence/absence of native predators. We found that leachates of eucalypt leaf litter reduced amphibian development and growth, compromised their antipredator responses and altered their metabolic rate. Increased temperature itself also posed serious alterations on development, growth, antioxidant ability and the immune status of tadpoles. However, the combined effects of eucalypt leaf litter and increased temperature were additive, not synergistic. Therefore, we show that non-lethal levels of a globally spread disruptor such as leachates from eucalypt leaf litter can seriously impact the life history and physiology of native amphibian populations. This study highlights the need to evaluate the status of wild populations exposed to human activities even if not at an obvious immediate risk of extinction, based on reliable stress markers, in order to anticipate demographic declines that may be hard to reverse once started. Replacing eucalypt plantations with native trees in protected areas would help improving the health of local amphibian larvae. In zones of economic interest, we would recommend providing patches of native vegetation around ponds and removing eucalypt leaf litter from pond basins during their dry phase.

## Teaser

Eucalypts, extensively used in tree plantations, have detrimental effects on tadpoles. When in ponds with eucalypt leaf litter, tadpoles reduce growth rate, have their predator detecting mechanism impaired and alter their metabolism. These effects are intensified at higher temperature, an additional cause for concern given current trends in climatic conditions.

## Introduction

Human activities over the last three centuries have caused grave perturbations worldwide resulting in temperature increase, desertification, forest plantations, urbanization and diminished water resources (Brook *et al.*, 2008; Eisenhauer *et al.*, 2012; Haddeland *et al.*, 2014). Such transformations have pushed so many species to the brink of extinction that they can be considered to be causing the sixth mass extinction (Leakey and Lewin, 1996; Ceballos *et al.*, 2017). In this scenario, even species currently considered to be at low extinction risk often show signs of steep population declines (Ceballos *et al.*, 2017). In fact, threats to biodiversity such as resource overexploitation, invasive species, or climate warming have increased markedly during the last decades (Butchart *et al.*, 2010).

Human perturbations often alter organisms’ development, growth, phenology, activity and/or fecundity hence resulting in poorer performance and reduced fitness (Parmesan, 2006; Helmuth, 2009). Such alterations at the organismal level can translate into changes in life-history traits (Deutsch *et al.*, 2008; Somero, 2012) and disrupt networks of ecological interactions (Tylianakis *et al.*, 2008). Landscape modification is one of the major aspects of global change. In addition to extensive urbanization, natural vegetation has been extensively logged or transformed. Across the globe, 7% of the total forested area has been transformed into forest plantations, ~20% of which consists of non-native species (FAO, 2015; Payn *et al.*, 2015). Reforestation with non-native plantations constitutes a sudden and drastic alteration of the environment. In response to these changes, local species with high mobility can migrate to more suitable habitats, but species with high phylopatry or little vagility will necessarily rely on plastic adjustments of their phenotype, like behavioral shifts or physiological adjustments to respond to the environmental modifications (Scheiner, 2016; Edelaar *et al.*, 2017).

Here we study the consequences for amphibian larvae of exposure to eucalyptus leaf litter, in terms of their life history, behavior and physiology, taking into consideration two other major ecological factors: temperature and predator presence. Amphibians constitute the most vulnerable group of vertebrates in the current global biodiversity crisis (Ducatez *et al.*, 2016), as 41% of their species are threatened (Hoffmann *et al.*, 2010) and >70% are in decline (Stuart *et al.*, 2004; Monastersky, 2014). Although causes producing amphibian declines are complex, habitat alteration, emergent diseases and invasive species seem to be its main causes (Ducatez *et al.*, 2016). The recurrent use of exotic plant species for reforestation or forest plantations have extensively modified both terrestrial and aquatic ecosystems (Charles and Dukes, 2008; Vilà *et al.*, 2011), both of which are of primary importance to amphibians. Specifically, eucalypt (*Eucalyptus* spp.) plantations are common worldwide, occupying an estimated area of >19 million ha across five continents (~1.31% of the total land coverage; Iglesias-Trabado and Wilstermann, 2008). In the Iberian Peninsula, eucalypt plantations are mainly located in the north and southwest and cover >1.4 million ha, representing 2.4% of the peninsula’s surface, 7% of eucalypt plantations worldwide (Greenpeace, 2011). In particular, eucalypt plantations in the Iberian Peninsula represent 53% of the world’s plantations for a specific species, *Eucalyptus globulus* (Greenpeace, 2011). *Eucalyptus* trees are characterized by fast growth and high adaptability to multiple environments, which facilitate their quick occupancy of ample areas. The introduction of these species has brought about negative local effects such as soil degradation, declining groundwater level, or decreased biodiversity (Foley *et al.*, 2005), including amphibian biodiversity (Vallan, 2002). Eucalypts produce leachates that are released into the ponds through its decaying leaf litter. Such eucalypt leachates can alter ponds’ water composition, litter dynamics, the structure of macroinvertebrate communities and the reproductive success of fish (Abelho and Graça, 1996; Morrongiello *et al.*, 2011). In this line, some previous studies have assessed the effects of exotic plants on amphibian performance, behavior, or predator detection (Maerz *et al.*, 2005; Martin and Murray, 2011; Watling *et al.*, 2011a; Iglesias-Carrasco *et al.*, 2017a), although the literature in this area is still sparse (Maerz *et al.*, 2005; Martin and Murray, 2011).

Surprisingly, despite the large extent of eucalypt reforestations worldwide, the effects of eucalypt plantations on aquatic vertebrates (and particularly on amphibians) have received very little attention (but see Iglesias-Carrasco *et al.*, 2016 and Iglesias-Carrasco *et al.*, 2017a). Although it has recently been shown that eucalypt leaf litter can impair mate and predator recognition in adult newts (Iglesias-Carrasco *et al.*, 2017b), the extent to which it also affects growth, development or physiology of amphibian larvae is virtually unknown. To this end, we designed an experiment in which larvae of the European common frog (*Rana temporaria*) were exposed to either eucalypt or native oak leaf litter under laboratory conditions. Moreover, since the effect of non-native plantations is likely to interact in natural conditions with both biotic and abiotic factors that can mitigate or exacerbate its consequences, we studied the effects of eucalypt in combination with different temperatures and the presence/absence of predators. Predictions of global warming led us to test the effects of eucalypt leaf litter also at a warmer temperature. Moreover, local predators are also likely to stay in ponds after the reforestation and it is unclear how predator cues and non-native leaf litter would combine to affect tadpole physiology and life histories. We hypothesized that eucalypt leaf litter would restrict larval growth and slow down development, whether directly due to the allelopathic compounds released into the water or indirectly through alterations in periphyton growth, for instance. We also hypothesized that exposure to eucalypt allelopathic compounds could have detrimental physiological consequences like other chemical pollutants do (Marquis *et al.*, 2009; Burraco *et al.*, 2013; Burraco and Gomez-Mestre, 2016), potentially altering metabolic rate, causing oxidative stress, or challenging their immune status. In addition, temperature variation may modulate eucalypt effects, as warm temperatures could impose additional stress to that caused by eucalypt, but low temperatures would also prolong the exposure of tadpoles to eucalypt by slowing down development and delaying metamorphosis. Moreover, alterations of the pond’s chemosphere with pollutants can interfere the ability of amphibian larvae to respond to predators (Polo-Cavia *et al.*, 2016) and eucalypt leaf litter is known to substantially modify the water’s chemosphere (Abelho and Graça, 1996). Inhibition of detection of predator cues would cause higher risk of predation, especially if the predators are visually oriented and do not depend themselves on chemical cues to detect their prey. Interactions among the studied factors would give us a more complete picture of the possible effects of eucalypt on amphibian larvae, whether it reduces their survival odds in the short term, or if it imposes a physiological toll of foreseeable longer-term consequences.

## Material and methods

### Study system and sampling

*Rana temporaria* is distributed throughout most of Europe. In the Iberian Peninsula, it mainly occupies forests located in the northwest of Spain and its tadpoles often face native predators such as dragonfly nymphs (*Aeshna* sp.), used in this study. We collected six clutches of *R. temporaria* and ~40 dragonfly nymphs in different ponds in northern Spain (Álava, Spain). All clutches were collected on the same day and in similar development stages, i.e. between Gosner stages five and seven (Gosner, 1960). We also collected both oak (*Quercus robur*) and eucalypt (*Eucalyptus globulus*) leaf litter at the same locations. We collected oak and eucalypt leaves that had recently fallen to the ground and hence corresponded to the top-most layer of litter in small 20 m^2^ patches within each forest. Leaves were dried immediately at room temperature (20 °C) for 72 h. Each clutch was introduced in a 3 L bucket whereas predators were individualized in 1 L buckets, and we immediately transported them to Doñana Biological Station (Seville, Spain). Upon arrival, we divided each clutch into two approximately equal portions and they were placed in 10-L buckets filled with carbon-filtered dechlorinated tap water, assigning each clutch portion to a different walk-in chamber set-up at two constant temperatures, either 15 or 20°C with a 12:12 light–dark cycle. Dragonflies were kept in 1-L buckets with artificial plants for perching and we randomly assigned one dragonfly to those treatments including predators.

### Experimental set-up

To test for the effect that the type of leaf litter, temperature and predators and also the effect of the interaction between these three factors on the life-history traits and physiology of *R. temporaria* tadpoles we designed a 2 × 2 × 2 experiment including: leaf litter type (*Quercus robur* or *Eucalyptus globulus*), temperature (15 or 20°C) and predators (present or absent). Our experiment thus consisted of eight treatments, each replicated nine times for a total of 72 experimental units. Each experimental unit consisted of 41 cm × 34 cm × 26 cm rectangular plastic containers filled with 30 L of carbon-filtered dechlorinated tap water. A constant air flow was added to each container with an aerator to prevent reduced levels of dissolved oxygen due to decaying leaf litter and other organic matter. Two days before the experiment started, we added 0.5 g/l of either dried eucalypt or oak leaves (following Maerz *et al.*, 2005) to each 30 L container (i.e. 15 g of leaves per container) to allow leaves to release leachates. Once hatched, we pooled tadpoles across all clutches together and randomly assigned 15 to each experimental container. Tadpoles were fed *ad libitum* with rabbit chow every third day. We used non-supplemented rabbit chow, mainly consisting in sun-dried alfalfa. All containers included a suspended plastic cup (250 mL) with a mesh screen bottom that allowed water flow and cue diffusion, and we added one predator in those containers assigned to predator treatments. Dragonfly nymphs were fed with one *R. temporaria* tadpole three times a week in their cup to ensure the release of both kairomones and conspecific alarm cues. All tadpoles from each tank except two were raised until metamorphosis, recording the date of tail resorption (Gosner stage 46; Gosner, 1960) as indicator of time to metamorphosis and weighing them to the nearest 0.1 mg on an electronic balance (CP324S, Sartorius). Time to and mass at metamorphosis was hence recorded, and growth rate was estimated as log(mass)-log(time to metamorphosis). Two randomly chosen tadpoles were collected after 21 days from the onset of the experiment: one being assigned to standard metabolic rate (SMR) and immune status determination assays and the second one to oxidative stress analyses.

### Behavioral assay

We quantified tadpole activity at day 20 from the onset of the experiment using repeated scans for recording the number of visible and active (swimming or feeding) tadpoles in each container (Relyea, 2004). A single observer obtained the average number of visible and/or active tadpoles by completing five scans in two hours. All the scans were performed from 10 to 12 am on a single day, the 20th from the beginning of the experiment, to avoid potential confounding effects of day and/hour on tadpole behavior. The five scans were done consecutively, always starting from the same tank to ensure that the interval between one scan and the next was similar for all tanks, ~25 min overall. Since tank position was randomized, scanning of consecutive tanks resulted in random order observations according to treatment. To avoid disturbing tadpole behavior, the observer approached the tank slowly and performed the scans at least from a 1-m distance. We did not observe changes in tadpole behavior (e.g. halted feeding and swim bursts into refugia) as the observer approached the tank.

### Standard metabolic rate

To quantify SMR we used an aquatic flow-through respirometer. Ten optical sensors (optodes) were mounted at the entrance and exit of five independent plexiglass chambers (44 mm in diameter × 163 mm in length) and connected to an oxymeter (Oxy 10-PreSens, Germany). We haphazardly removed one tadpole per experimental unit and introduced individually in a plexiglass chamber at a time, taking a few seconds. We obtained five independent measurements of oxygen consumption (mg/l) in each round. SMR measurements were carried out using water at each of the experimental temperatures (15 and 20 °C), as temperature is an important factor modulating metabolic rate. Nevertheless, all assays were conducted using clean dechlorinated water, to prevent the coloring effect of leaf litter leachates interfering with the optodes of the respirometer. We recorded SMR during 20 min but discarded the first five minutes as acclimation period (Burraco and Gomez-Mestre, 2016). SMR was calculated as VO_2_ = Vw · ∆Cw; where VO_2_ is the rate of oxygen consumption (in mg/h; Álvarez *et al.*, 2006), Vw is the flow rate through the chamber (l/h) and ∆Cw is the instantaneous difference in oxygen concentration between the inflow and outflow water. Immediately after SMR determination, we weighed each individual to the nearest 0.1 mg to use weight as covariate in the analyses and proceeded to evaluate their immune response (see below).

### Immune response

We estimated the absolute count of leukocytes and the relative proportion of their different cell types through flow cytometry, a technique recently validated for amphibians (Burraco *et al.*, 2017a; see Supplementary material S1). To that end we haphazardly selected one individual from each tank (i.e. *N* = 9 individuals per treatment), anesthetized it with MS-222, obtained a small blood sample via cardiac puncture with a 29-G syringe (BD Micro-Fine U-100 0.5 mL) and collected it in heparinized tubes. We differentiated four types of blood cells based on their size and complexity, following the gating strategy described in Burraco *et al.* (2017a): erythrocytes, lymphocytes, granulocytes and monocytes. However, because monocytes are very scarce in amphibian blood (Davis *et al.*, 2008), we discarded this cell type from further analyses. We used granulocyte-to-lymphocyte ratio and the count of both granulocytes and lymphocytes in relation to the total number of erythrocytes as an assessment of the immune state.

### Oxidative stress

We measured the activity of four antioxidant enzymes: catalase (CAT), superoxide dismutase (SOD), glutathione peroxidase (GPx) and glutathione reductase (GR). We also quantified the concentration of malondialdehyde (MDA), which indicated damages in cellular lipids, and the reduced-to-oxidized glutathione ratio (GSH-to-GSSG) as an indicator of cellular antioxidant capacity.

One tadpole per tank (*N* = 9 individuals/treatment) was euthanized with MS-222 (SIGMA) and then snap frozen in liquid nitrogen and stored at −80°C until assayed. Prior to the assays, tadpoles were eviscerated to avoid possible interference of gut content on biochemical determinations. Eviscerated tadpoles were immersed in a buffered solution that inhibits proteolysis (Burraco *et al.*, 2013) in a proportion of 1 g tissue in 4 ml of homogenization buffer. Samples were homogenized individually using a Miccra homogenizer (Miccra D-1). Finally, we centrifuged the homogenates at 14 000 rpm for 30 min at 4 °C and quantified total protein content by standard Bradford’s procedure (Bradford, 1976). See Supplementary material S1 for details on oxidative stress protocols.

### Statistics

All statistical analyses were run in R, version 3.3.1 (R Development Core Team). We checked parametric assumptions using Kolgomorov-Smirnov tests for assessing data normality using the function *lillie.test* (‘nortest’ package, version 1.0-2) and Breusch–Pagan tests for assessing homoscedasticity using the function *bptest* (‘lmtest’ package, version 0.9-35). We fitted linear models using the function *lm* (‘stats’ package, version 3.5.0) and both linear and generalized mixed models using the function *lmer* and *glmer* (‘lme4’ package, version 1.1-7). We ran linear models for variables for which we had a single data point per tank, as number of active tadpoles, SMR, antioxidant activity, MDA and glutathione and generalized linear models using a binomial error distribution for behavior data. We fitted linear mixed models for variables with Gaussian error distributions such as larval period, body mass and growth rate; and we fitted generalized mixed models for survival and proportion of leukocytes, using a binomial error distribution. We included experimental tank as a random factor in the mixed models. Days to metamorphosis, body mass, oxygen consumption and MDA were log-transformed to meet parametric assumptions. We estimated growth rate as log (body mass at metamorphosis)—log (days to reach metamorphosis). We used log (body mass) as a covariate in models testing for variation in SMR. For *post hoc* pairwise comparisons we run Tukey-tests. We used principal components analysis (PCA) to examine variation in antioxidant activities and possible redundancy of variables. The ratio GSH-to-GSSG was estimated as log(GSH)—log(GSSG).

## Results

Survival at metamorphosis was relatively high throughout the experiment (68.3%). We found no effect of leaf litter type or predator presence on tadpole survival, nor did we find evidence for interactions among factors (all *P* > 0.352). Survival was only marginally affected by temperature, as tadpoles at high temperature had on average 47.75% lower chances of surviving than those at low temperature (*χ*^2^ = 3.69, *P* = 0.055).

### Developmental and growth responses

**Figure 1:**
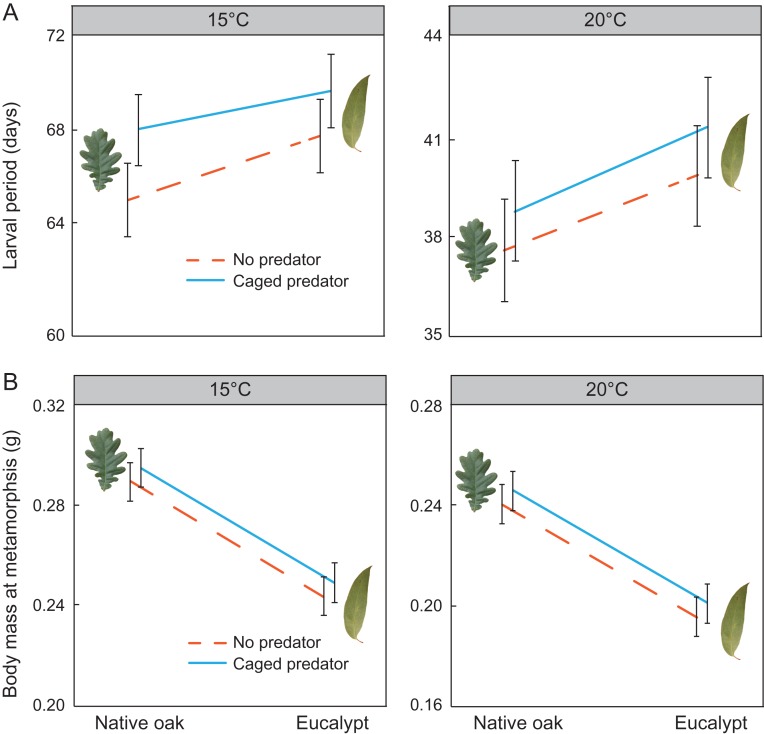
The effect of leaf litter (native oak or eucalypt) and predator (absent or present) on larval period (**A**) and body mass at metamorphosis (**B**) in *Rana temporaria* metamorphs reared at 15 and 20°C. Data are estimated marginal mean ± standard error. Lines indicate the presence (blue solid line) and absence (dashed orange line) of predators. Note that *y*-axis scale varies for each graph

Tadpoles exposed to eucalypt leaf litter metamorphosed on average 2.27 days later (4.16% of total larval period) than those exposed to oak leaf litter (*χ*^2^ = 5.54, *P* = 0.019; Fig. 1A). As expected, tadpoles reared at 20°C metamorphosed faster (71.72% faster on average) than tadpoles at 15°C (*χ*^2^ = 186.20, *P* < 0.001; Fig. 1A). Predator presence tended to delay metamorphosis in *R. temporaria* tadpoles, but this change was marginally non-significant (*χ*^2^ = 3.48, *P* = 0.062). We found no significant two-way interactions among leaf litter type, temperature and predator presence and no significant three-way interaction either, all *P*-values for the interactions being >0.121.

Eucalypt life-litter reduced tadpole growth rate by 21.19% on average (*χ*^2^ = 30.25, *P* < 0.001) and body mass at metamorphosis by 18.14% (*χ*^2^ = 30.60, *P* < 0.001; Fig. 1B) compared to tadpoles raised with oak life-litter. Low temperature also slowed down growth, so that tadpoles at 15°C had 28.84% lower growth rate (*χ*^2^ = 51.40, *P* < 0.001), although they resulted in 22.63% heavier metamorphs (*χ*^2^ = 37.05, *P* < 0.001, Fig. 1B). Predator presence did not affect tadpole growth rate or body mass (*χ*^2^ = 0.07, *P* = 0.787 and *χ*^2^ = 0.63, *P* = 0.428, respectively). We found no evidence for neither three- nor two-way interactions among leaf litter type, temperature and predator presence (all *P* > 0.165).

Body mass at metamorphosis was not affected by triple or double interactions (all *P* > 0.090). However, all factors affected larval body mass so that the presence of predators increased it by 2.28% (*χ*^2^ = 708.18, *P* < 0.001), low temperature increased it by 22.63% (*χ*^2^ = 744.6, *P* < 0.001) and eucalypt leaf litter reduced it by 20.67% (*χ*^2^ = 738.2, *P* < 0.001).

### Behavioral responses

**Figure 2:**
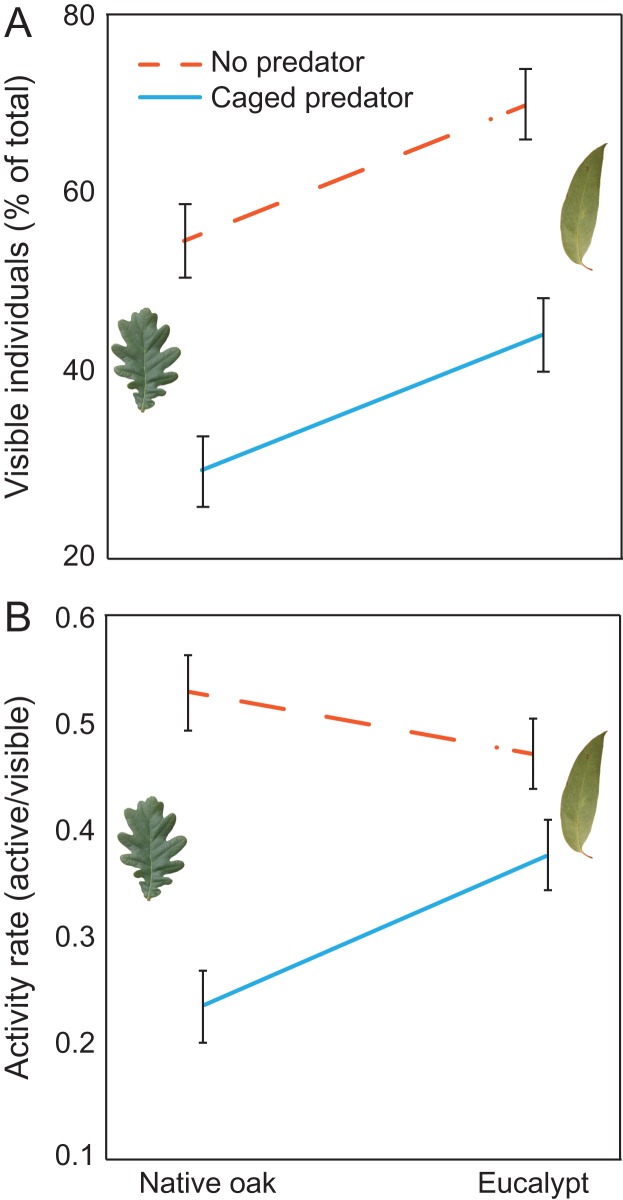
The effect of leaf litter (native oak or eucalypt) and predator (absent or present) on hiding behavior (**A**) and activity (**B**) in *Rana temporaria* larvae. Data are estimated marginal mean ± standard error. Lines indicate the presence (blue solid line) and absence (dashed orange line) of predators

Tadpoles exposed to eucalypt leaf litter increased by 26.33% the time they were visible (i.e. not hiding amongst leaf litter) compared to tadpoles with oak leaf litter (*χ*^2^ = 38.60, *P* < 0.001; Fig. 2A). Tadpoles were significantly less often visible at high temperature than at low temperature (24.12% less visible on average; *χ*^2^ = 14.20, *P* = 0.002) and in the presence of predators than in their absence (57.76% less visible on average with predators; *χ*^2^ = 70.43, *P* < 0.0001; Fig. 2A). No interactions among factors were detected (all *P* > 0.130). Regarding general tadpole activity, we found that tadpoles were less active in the presence of predators (38.78% less active on average; Fig. 2B). However, their behavioral response varied depending on the type of leaf litter, as indicated by a leaf litter by predator interaction (*χ*^2^ = 5.10, *P* = 0.024; Fig. 2B). Thus, tadpoles reduced their activity to a greater extent (56.05% on average) with oak leaf litter than when exposed to eucalypt leaf litter (20.06% activity reduction on average; Fig. 2B). Temperature itself did not alter tadpole activity (*χ*^2^ = 0.34, *P* = 0.559). No other interaction was found to be significant (all *P* > 0.392).

### Standard metabolic rate

**Figure 3:**
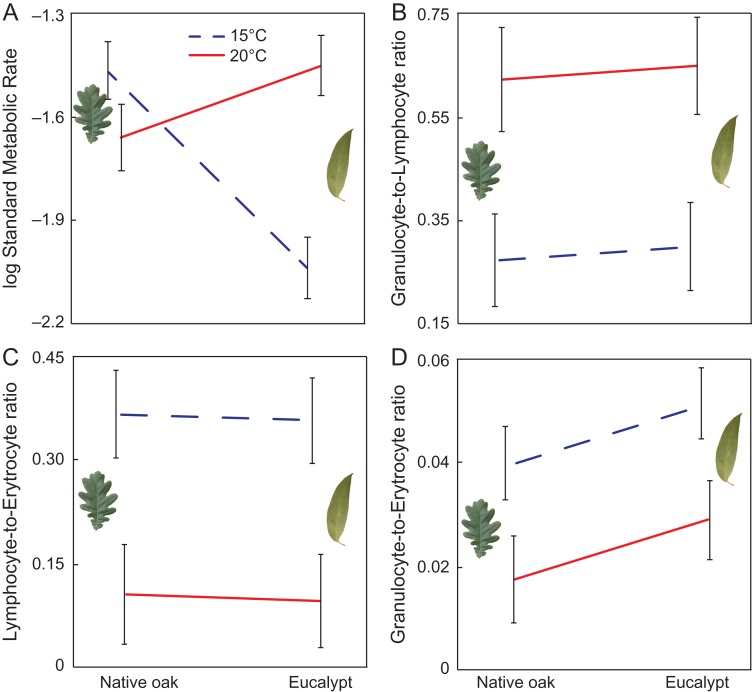
The effect of leaf litter (native oak or eucalypt) and temperature (15°C indicated by purple dashed line or 20°C indicated by solid red line) on standard metabolic rate (**A**), granulocyte-to-lymphocyte ratio (**B**), lymphocyte-to-erythrocyte ratio (**C**) and granulocyte-to-erythrocyte ratio (**D**) in *Rana temporaria* larvae. Data are estimated marginal mean ± standard error. In the case of standard metabolic rate, the models included body mass as a covariate

Leaf litter type affected the metabolic rate of *R. temporaria* tadpoles, although the effect was temperature dependent, as indicated by the interaction between leaf litter and temperature (*χ*^2^ = 19.49, *P* < 0.001; Fig. 3A). Tadpoles exposed to eucalypt leaf litter at 15°C consumed on average 28.37% less oxygen than tadpoles at the same temperature but with oak leaf litter (*post hoc* test = 0.004; Fig. 3A). However, no leaf litter type effect on metabolic rate was detected at 20°C (*post hoc test* = 0.363). Overall, high temperature increased SMR of tadpoles by an average 12.60% (*χ*^2^ = 5.11, *P* = 0.028; Fig. 3A). Predator cues, however, did not alter tadpoles’ metabolic rate (*χ*^2^ = 0.01, *P* = 0.953). We found no other significant interactions, whether two- or three-way (all other *P* > 0.411).

### Immune response

We found no effect of leaf litter type or predator presence on immune status of the tadpoles, nor did we find evidence for interactions among factors. Immune status was nonetheless affected by temperature, as tadpoles at 20°C increased their granulocyte-to-lymphocyte ratio 1.10-fold compared to tadpoles at 15°C (χ*^2^* = 13.56, *P* < 0.001; Fig. 3B). Moreover, tadpoles at 20°C experienced low lymphocyte count (lymphopenia) compared to tadpoles at 15°C (*χ*^2^ = 8.68, *P <* 0.001) by 72.11% on average (Fig. 3C). Similarly, tadpoles at 20°C experienced a decrease in their absolute granulocyte count (granulopenia) by 49.31% compared with those at 15°C (*χ*^2^ = 7.72, *P* = 0.005; Fig. 3D) whereas eucalypt exposure produced a marginally non-significant increase of the absolute granulocyte count (*χ*^2^ = 3.49, *P* = 0.062).

### Oxidative stress

**Figure 4:**
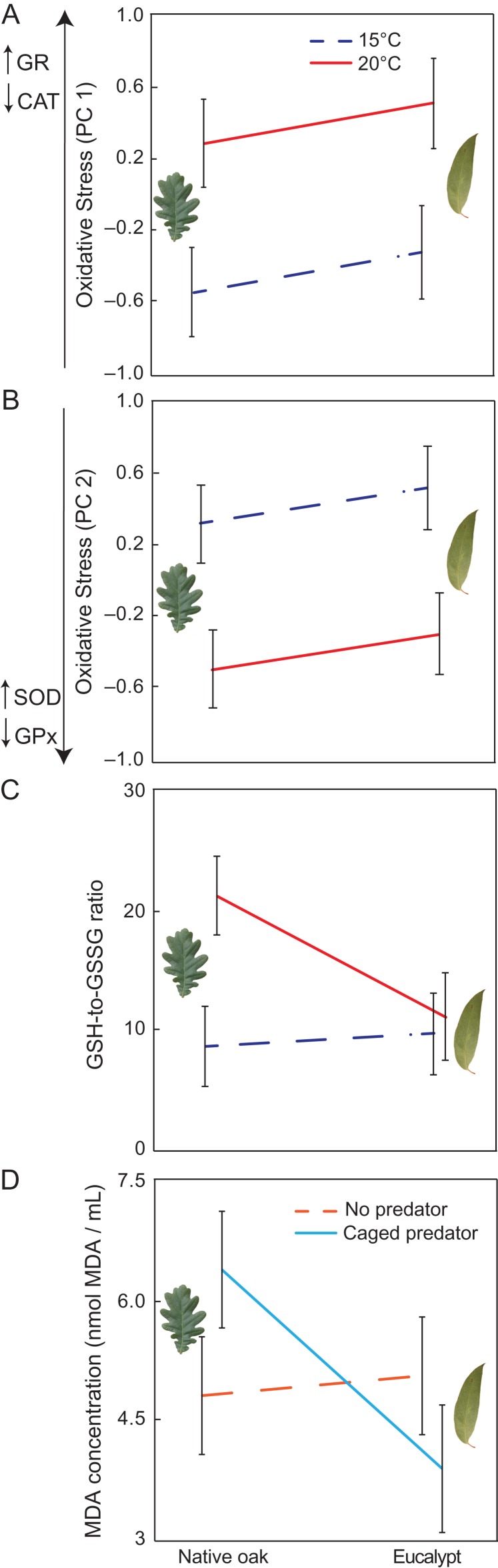
The effect of leaf litter (native oak or eucalypt) and temperature (15°C indicated by blue dashed line or 20°C indicated by solid red line) on: (**A**) the first and second (**B**) components of principal component analysis from antioxidant activities data, (**C**) reduced-to-oxidized glutathione (GSH-to-GSSG) ratio. Positive values in (A) indicate increases in the activity of glutathione reductase and reductions in the activity of catalase. Negative values in (B) indicate increases in the activity of superoxide dismutase and decreases in the activity of glutathione peroxidase. (**D**) Effect of leaf litter type and predator presence or absence in malondialdehyde levels (in nmol/ml) in *Rana temporaria* larvae. Data are estimated marginal mean ± standard error.

Antioxidant activity values of catalase, glutathione reductase, GPx and SOD reported four principal components (PCs) of which PC1 and PC2 explained the major differences on antioxidant activities (40.7 and 32.9%, respectively; see Supplementary material S2). Positive values on PC1 mainly indicated increased GR activity and decreased CAT activity; whereas positive values on PC2 explained increased GPx activity and decreased SOD activity (Supplementary material S2). Activity of antioxidant enzymes significantly varied across temperature treatments according to differences in PC1 and PC2 (*χ*^2^ = 8.20, *P* = 0.006 and *χ*^2^ = 9.80, *P* = 0.003, respectively). Changes in PC1 indicated that tadpoles at 20°C mainly showed higher GR activity and lower CAT activity (Fig. 4A), whereas changes in PC2 indicated that tadpoles at 20°C showed higher GPX activity and lower SOD activity than tadpoles at 15°C (Fig. 4B). We found no effect of leaf litter type or predator presence on antioxidant enzyme activity and no evidence for interactions among factors (all *P* > 0.225). In relation to lipid peroxidation through estimates of MDA levels, we detected, however, a significant leaf litter type by predator interaction in MDA levels (*χ*^2^ = 34.64, *P* = 0.035; Fig. 4D), showing that lipid peroxidation was higher in tadpoles exposed to oak leaves when predators were present in comparison to tadpoles exposed to eucalypt litter. We found no other significant interactions regarding MDA levels (all *P* > 0.233). Finally, the GSH-to-GSSG ratio was not affected by leaf litter type or predator presence, whereas temperature had a significant effect on it (44.09% increase on average between temperatures; *χ*^2^ = 4.640, *P* = 0.0348; Fig. 4 C). No significant interactions were found for cellular antioxidant ability (all *P* > 0.079).

## Discussion

Impoverished individual performance due to human–driven disturbances often scale up to declines in natural populations (Harrison *et al.*, 2011; Paquette *et al.*, 2014). Detecting reductions in performance and physiological alterations would facilitate a rapid diagnose of the impact of global changes on the homeostasis of local populations, even below lethal levels (ViBlanc, 2018). The detection of such perturbations would allow customized designs of environmental protection policies that could help preventing subsequent population declines. Here, we find that a common but underestimated disruptor, such as habitat transformation or reforestation with non-native species, can have direct detrimental consequences for amphibian larvae.

Previous studies have described negative effects of allelopathic substances produced by non-native plants on life-history traits of amphibian larvae (Watling *et al.*, 2011a; 2011b; Perez *et al.*, 2013), although the extent of these effects seems to be both plant and amphibian species-dependent (Maerz *et al.*, 2005, Cohen *et al.*, 2012). In our experiment, eucalypt leaf litter slowed down development in *R. temporaria*. Developmental timing in anurans is mainly mediated by the combined effect of thyroid hormone and corticosterone (Denver, 2009). Increased production of these hormones is common in amphibians exposed to risky environmental conditions such as desiccation, salinity or herbicide (Gomez-Mestre *et al.*, 2013; Burraco and Gomez-Mestre, 2016). Slow development may result in larger or smaller metamorphs depending on the relative effect of environmental factors on growth and differentiation (Gomez-Mestre *et al.*, 2010; Burraco *et al.*, 2017b). Here we show that chronic exposure to eucalypt leaf litter also reduced growth rate, so that exposed tadpoles had both longer larval periods and smaller size at metamorphosis. Eucalypt leaf litter may reduce nutrient levels in the water (Molinero and Pozo, 2004), but as we supplemented tadpoles with *ad libitum* rabbit chow, reductions in growth in our experiment were likely caused directly by allelopathic substances released by eucalypt litter rather than indirectly from reduced periphyton growth. Reductions in size at metamorphosis like those observed here are critical because in species with complex life-cycles, body mass at ontogenetic switch-points largely determines the odds of survival in subsequent life stages (Pechenik *et al.*, 1998; Gomez-Mestre and Tejedo, 2003; Cabrera-Guzman *et al.*, 2013).

Water containing leachates from eucalypt leaf litter interfered with the behavioral responses of tadpoles to predators. Under predation risk, larvae of most amphibian species show adaptive behavioral responses consisting in reduced activity rate (Werner and Anholt, 1996). We observed that *R. temporaria* larvae responded to the presence of predator cues when reared in water containing oak leaf litter, but failed to reduce activity when water contained eucalypt leaf litter. Also, tadpoles were more visible when reared in water containing eucalypt leaves, despite the presence of predators. Hiding under leaf litter is also an effective antipredator strategy (Van Buskirk, 2001; Hossie and Murray, 2010), so failing to find refuge in the presence of predators would substantially reduce tadpoles’ odds of survival. This failure to respond to predator cues in tadpoles exposed to eucalypt leaf litter suggests that eucalypt compounds inhibit predator recognition. Alternatively, tadpoles could be recognizing predator cues but not being able to reduce their activity rate to compensate the reduction in growth caused by the eucalypt leachate. However, the indications of physiological stress were not severe, as eucalypt leaf litter had scarcely any effect on the immune status or oxidative stress of larvae. Therefore, we consider that eucalypt leachates were more likely interfering with predator recognition and not necessarily impairing their ability to respond, as was the case in other cases of non-native plant leachates (Hickman and Watling, 2014; Iglesias-Carrasco *et al.*, 2017b), or more generally with other alterations of the ponds’ chemosphere (Polo-Cavia *et al.*, 2016).

Eucalypt leaf litter also affected tadpole metabolic rate. Larvae reared with eucalypt leaf litter at 15°C reduced their metabolic rate in comparison to those exposed to oak leaves. Such decrease in oxygen consumption may be due to toxic effects of eucalypt leachates, in accord with the observed reduced growth. The effect of eucalypt on metabolic rate seemed to be offset by a warmer temperature, as it was not detectable at 20°C. Changes in metabolic rate often depend on the nature and type of the stressful environment. For instance, elevated concentration of trace elements may cause increased metabolic rate in many species (e.g. Rowe *et al.*, 2001; Pistole *et al.*, 2008) whereas other disruptors such as hydrogen peroxide, food deprivation, or even predator presence can induce lower metabolic rates (Abele-Oeschger *et al.*, 1997; Renault *et al.*, 2003; Steiner and Van Buskirk, 2009). Such alterations of metabolism, together with developmental, growth and behavioral disturbances detected in larvae reared in eucalypt leaf litter, are clear signs of toxicity. Despite the observed alterations in growth, development and behavior, we only found minimal effects of eucalypt leachates on oxidative stress and immune system of *R. temporaria* tadpoles. Regarding the oxidative status, levels of antioxidant enzymes and the proportion of reduced glutathione remained unaltered by eucalypt leachates. MDA levels instead were higher in tadpoles reared with oak leaves and predator presence. High MDA levels can indicate damages in lipids but can also be confounded by larger fat content in those individuals (Pérez-Rodríguez *et al.*, 2015), and tadpoles in the presence of predators and oak leaf litter were the largest and also less active, so they were likely to also have greater fat bodies.

Temperature can vary substantially among eucalypt plantations even within small geographical areas such as elevation or orientation, and even within a specific site global warming is expected to increase temperatures locally. Therefore, it is of interest to understand the combined effect of eucalypt and temperature on amphibian larvae. As expected, increased temperature accelerated larval development while reducing body mass at metamorphosis (Álvarez and Nicieza, 2002; Gomez-Mestre *et al.*, 2010) and also altered larval metabolism. Particularly, increased temperatures induced increases in the activity of some antioxidant enzymes (GR and SOD). Variations in antioxidant activities in organisms facing temperature increases have been extensively addressed in plants (Sairam *et al.*, 1997; Gill and Tuteja, 2010) and to a lesser extent in vertebrates (Selman *et al.*, 2000; Madeira *et al.*, 2013; Burraco and Gomez-Mestre, 2016). Prolonged oxidative stress may accelerate cellular death (Ott *et al.*, 2007; Holmström and Finkel, 2014) and organisms’ senescence (Speakman and Selman, 2011; Costantini, 2014). In addition, oxidative stress during early life stages can shape later stages (Metcalfe and Alonso-Alvarez, 2010) and explain differences in developmental adaptive responses among populations (Burraco *et al.*, 2017c). Also, larvae exposed to high temperature had higher GSH-to-GSSG ratio, indicating higher antioxidant capacity of organisms, as GSH is a vital intra and extracellular antioxidant (Zitka *et al.*, 2012). GSH can be overproduced under thermal stress because it facilitates hepatic GSH release (Mitchell *et al.*, 1983; Mahmoud and Edens, 2003). Therefore, we can conclude high temperature induces cellular oxidative stress likely caused by increased metabolism, although it did not reduce antioxidant ability or produce damages in lipids. Finally, at higher temperature tadpoles showed signs of slight immunosuppression, as the ratio of granulocyte-to-lymphocyte ratio was higher, possibly due to higher glucocorticoid secretion (Davis *et al.*, 2008; Burraco *et al.*, 2017a). However, the effect of temperature was largely additive with the effect of eucalypt, as there were no significant interactions between the two factors for the most part.

## Conclusions

Leachates from eucalypt leaf litter cause delayed development, reduced growth rate and size at metamorphosis, failure to recognize predator cues and mild physiological stress in *R. temporaria* tadpoles. These deleterious effects were exacerbated by increased water temperature, although in an additive, not a synergistic way. In view of these results, we recommend replace eucalypt plantations by native ones, at least in protected areas, and remove eucalypt leaf litter from the basin of temporary ponds during their dry phase in order to minimize the impact of eucalypt forest on amphibian populations.

## Supplementary Material

Supplementary Data

Supplementary Data

Supplementary Data

## Data accessibility

We will make the original data available in Dryad upon acceptance of the MS
