# Supplementary material for: Eucalypt leaf litter impairs growth and development of amphibian larvae, inhibits their antipredator responses and alters their physiology
Source: Conserv Physiol. 2018 Dec 10;6(1):coy066. doi: 10.1093/conphys/coy066 (PMC6287674; doi:10.1093/conphys/coy066)
Supplement: Supplementary Data [file coy066_si2.docx]

**Supplementary material S2.**

| **Loading values** | PC1 | PC2 | PC3 | PC4 |
| --- | --- | --- | --- | --- |
| CAT | -0.5450558 | 0.2248428 | 0.805843397 | 0.05455574 |
| GR | 0.6466222 | 0.2804461 | 0.316119547 | 0.63505762 |
| GPx | 0.2538095 | 0.7627336 | -0.000873176 | -0.59482553 |
| SOD | 0.4694408 | -0.537618 | 0.500683622 | -0.48980412 |

| **Importance of components** | PC1 | PC2 | PC3 | PC4 |
| --- | --- | --- | --- | --- |
| Standard deviation | 1.2753 | 1.1475 | 0.8317 | 0.60439 |
| Proportion of Variance | 0.4066 | 0.3292 | 0.1729 | 0.09132 |
| Cumulative Proportion | 0.4066 | 0.7358 | 0.9087 | 1 |
