# Supplementary material for: Eucalypt leaf litter impairs growth and development of amphibian larvae, inhibits their antipredator responses and alters their physiology
Source: Conserv Physiol. 2018 Dec 10;6(1):coy066. doi: 10.1093/conphys/coy066 (PMC6287674; doi:10.1093/conphys/coy066)
Supplement: Supplementary Data [file coy066_supplementary_information_s1.docx]

**Supplementary Information S1**

**Oxidative Stress methodology**

Catalase activity was quantified following Cohen & Somerson (1969). Potassium permanganate (KMnO_4_) oxides and colors the substrate of the catalase, hydrogen peroxide (H_2_O_2_). That way KMnO_4_ is reduced, producing a red product. The absorbance of this product is read at a wavelength of 480 nm five minutes after the addition of KMnO_4_. We prepared standard curves of commercial CAT (SIGMA-60634) and expressed the catalase activity as U/mg of total proteins. The activity of superoxide dismutase was estimated spectrophotochemically by measuring the ferrocytochrome c inhibition rate, based on the presence of xanthine oxidase as the source of peroxide radicals (O_2_^-^). The unit of superoxide dismutase was defined as the amount of enzyme needed to inhibit the rate of reduction of cytochrome c by 50%, at 25 ºC and at a wavelength of 550 nm (McCord & Fridovich, 1969). To determine the glutathione reductase activity, we measured the decrease in absorbance at 340 nm derived from the NADPH oxidation, as described in Cribb, Leeder & Spielberg (1989). Finally, to quantify the activity of glutathione peroxidase we measured NADPH oxidation by reading absorbance at a wavelength of 340 nm (Paglia & Valentine, 1967).

One product of lipid peroxidation is malondialdehyde, which reacts with thiobarbituric acids generating a red product that absorbs light at 535 nm. Malondialdehyde concentration was quantified by subtracting blank values to the absorbance of each sample, and then compared to the calibration values. Finally, we quantified concentration of reduced and oxidized glutathione (GSH and GSSG, respectively) following the protocol developed by Galván et al. (2010).

**Leukocyte profile methodology**

We obtained blood via cardiac puncture with non-heparinized syringes (BD Micro-Fine insulin 29G 0.5 mL). Extracted blood was introduced in a heparinized tube kept on a layer of tissue paper over ice to prevent direct contact with ice hence avoiding hemolysis. We mixed by vortexing 1 µL of blood in 1 mL of Hanks’ balanced salt solution and with 1 µL of 3,3’-Dipentyloxacarbocyanine iodide (DiOC_5_(3), Sigma-Aldrich, St. Louis, MO) previously diluted 1:10 in absolute methanol. The reagent DiOC_5_(3) is a fluorescent lipophilic dye that is photostable when incorporated onto biological membranes, thus it can allow cell identification based on their size and complexity (Uchiyama et al. 2005). Immune cells abundance was determined two minutes after adding the DiOC_5_(3) with a flow cytometer (Guava Easy Cyte Plus, Guava Technologies).

**References**

Cohen, G., & Somerson, N. L. (1969). Catalase-aminotriazole method for measuring secretion of hydrogen peroxide by microorganisms. *Journal of bacteriology*, **98**, 543-546.

McCord, J. M. & Fridovich, I. (1969). Superoxide dismutase an enzymic function for erythrocuprein (hemocuprein). *Journal of Biological chemistry*, **244**, 6049-6055.

Cribb, A. E., Leeder, J. S. & Spielberg, S. P. (1989). Use of a microplate reader in an assay of glutathione reductase using 5, 5′-dithiobis (2-nitrobenzoic acid). *Analytical biochemistry*, **183**, 195-196.

Paglia, D. E. & Valentine, W. N. (1967). Studies on the quantitative and qualitative characterization of erythrocyte glutathione peroxidase. *The Journal of laboratory and clinical medicine*, **70**, 158-169.

Galván, I., Gangoso, L., Grande, J. M., Negro, J. J., Rodríguez, A., Figuerola, J. & Alonso-Alvarez, C. (2010). Antioxidant machinery differs between melanic and light nestlings of two polymorphic raptors. *PLoS One*, **5**, e13369.

Uchiyama, R., Moritomo, T., Kai, O., Uwatoko, K., Inoue, Y. & Nakanishi, T. (2005). Counting absolute number of lymphocytes in quail whole blood by flow cytometry. *Journal of veterinary medical science*, **67**, 441-444.
